# Fungi’s Swiss Army Knife: Pleiotropic Effect of Melanin in Fungal Pathogenesis during Cattle Mycosis

**DOI:** 10.3390/jof9090929

**Published:** 2023-09-15

**Authors:** Víctor Romero, Carolina Kalinhoff, Luis Rodrigo Saa, Aminael Sánchez

**Affiliations:** 1 Maestría en Biotecnología Agropecuaria, Universidad Técnica Particular de Loja, San Cayetano Alto, Calle París s/n, Loja 1101608, Ecuador; 2 Museo de Zoología, Universidad Técnica Particular de Loja, San Cayetano Alto, Calle París s/n, Loja 1101608, Ecuador; 3 Departamento de Ciencias Biológicas y Agropecuarias, Facultad de Ciencias Exactas y Naturales, Universidad Técnica Particular de Loja, San Cayetano Alto, Calle París s/n, Loja 1101608, Ecuador; cgkalinhoff@utpl.edu.ec (C.K.);

**Keywords:** beef and dairy livestock, bovine, evasion, fungal infection, homology, knowledge transfer, melanotic fungus, orthologous genes

## Abstract

Fungal threats to public health, food security, and biodiversity have escalated, with a significant rise in mycosis cases globally. Around 300 million people suffer from severe fungal diseases annually, while one-third of food crops are decimated by fungi. Vertebrate, including livestock, are also affected. Our limited understanding of fungal virulence mechanisms hampers our ability to prevent and treat cattle mycoses. Here we aim to bridge knowledge gaps in fungal virulence factors and the role of melanin in evading bovine immune responses. We investigate mycosis in bovines employing a PRISMA-based methodology, bioinformatics, and data mining techniques. Our analysis identified 107 fungal species causing mycoses, primarily within the Ascomycota division. *Candida*, *Aspergillus*, *Malassezia*, and *Trichophyton* were the most prevalent genera. Of these pathogens, 25% produce melanin. Further research is required to explore the involvement of melanin and develop intervention strategies. While the literature on melanin-mediated fungal evasion mechanisms in cattle is lacking, we successfully evaluated the transferability of immunological mechanisms from other model mammals through homology. Bioinformatics enables knowledge transfer and enhances our understanding of mycosis in cattle. This synthesis fills critical information gaps and paves the way for proposing biotechnological strategies to mitigate the impact of mycoses in cattle.

## 1. Introduction

Fungi have been shaping and influencing life on Earth for over a billion years [1]. Compared to drug-resistant bacterial infections or viral outbreaks, mycoses received relatively little attention, despite posing a significant threat to public health, food biosecurity, and biodiversity [2,3]. Worldwide, over 300 million people suffer from serious fungal diseases, and more than 1.6 million people die from severe fungal infections yearly [4]. Fungi and fungi-like microorganisms (e.g., Oomycetes) destroy a third of all food crops yearly, which would be sufficient to feed 600 million people.

Historically, we perceive the mycoses that occur in animals as low-prevalence diseases. However, the incidence of recalcitrant, recurrent, and massive mycoses has increased sharply during past decades [5]. For example, the discovery of independent strains of the human pathogen *Candida auris* simultaneously on three continents [6] could be the first documented example of an emerging fungal disease as a consequence of global warming [7].

Mycoses outbreaks are causing mass mortality in several non-human animal species. For example, chytridiomycosis, an infectious disease of amphibians caused by the chytrid fungi *Batrachochytrium dendrobatidis* and *B. salamandrivorans*, is considered the most dramatic disease-driven biodiversity decline ever recorded [8]. The nosemosis caused by *Nosema apis* and *N. ceranae* determine mass mortality in *Apis mellifera* bees [9,10]. Infectious fungal diseases contribute significantly to the extinction of chelonians, ranking among the top five causes of decline [11]. For example, *Fusarium solani* is responsible for mass mortalities by fusariosis in sea turtles’ eggs [12]. The fungal-like pathogen *Sphareothecum destruens* causes high mortality in some European fish populations [13]. Fungi can be infectious agents that attack mammals too, causing a plethora of diseases. Fungi cause the critical decrease of several bat populations through the white-nose syndrome caused by *Pseudogymnoascus destructan* [14].

This article focuses on mycoses that occur in mammals, particularly those reported in cattle. Mammal mycoses can range from superficial skin and mucosal infections to invasive or systemic mycoses that affect major internal organs. Systemic mycoses are less frequent than superficial mycoses and can be highly fatal, killing countless farmed and wild mammals [4]. Our limited understanding of the mechanisms involved in fungal pathogenesis and virulence hinder our ability to prevent, treat, and eradicate fungal diseases in cattle.

A growing body of evidence suggests that melanin plays a relevant role in fungal pathogenesis. It seems that melanin has a pleiotropic effect, participating in different evasion mechanisms that pathogenic fungi employ during infection to evade the immune response of their hosts [5]. The main objective of this research is to summarize the virulence factors associated with fungal pathogens and the relevance of melanin in the evasion of the cattle immune response by fungi. Based on an exhaustive bibliographic search, we provide an extensive reference list of the major fungal pathogens that infect cattle (*Bos taurus*), the main fungal diseases they cause, and their major virulence factors, including those in which melanin is involved. This review synthesizes evidence that can help fill the information gaps associated with this problem and lead to the proposal of biotechnological strategies that reduce the impacts of fungal mycoses in cattle.

## 2. Materials and Methods

The methodology used in this article was adapted from the Preferred Reporting Items for Systematic Reviews and Meta-Analyses (PRISMA) [15], but includes various bioinformatics and data mining procedures. We considered pathogenic fungi as those fungal species referred in the literature as mycosis-causing agents. We follow the taxonomic scheme of Mycobank [16,17]. The phylogenetic relationships between many Microsporidia and their inclusion within the kingdom of Fungi remain controversial [18]. However, we include Microsporidia in our study because infections caused by this group have historically been referred to as mycoses (i.e., Microsporidiosis) [19].

The analyzes were carried out using the R Studio user interface [20] and the following two R software packages [21]: 1.—‘litsearchr’ allows us to identify keywords without relying on a potentially biased set of pre-selected articles. This process is automated, fast, objective, reproducible, and easy to implement. ‘litsearchr’ is based on text mining and keyword co-occurrence networks [22]. ‘litsearchr’ also contains a set of functions to improve the efficiency of systematic reviews by automatically deduplicating and assembling results from separate databases. 2.—‘quanteda’ is a compilation of software packages for managing and analyzing textual data that allows natural language processing and transformation into structured text. Its capabilities match or exceed those provided by paid, proprietary end-user software applications [23].

### 2.1. Research Questions

The main research questions addressed in this review are: Q1.—Does research on cattle fungal diseases suffer the same general bias, receiving less attention than viral or bacterial diseases? Q2.—How many and which species of fungi are pathogens of cattle? Q3.—What are the mycoses caused by these pathogens? Q4.—What are the main factors that influence their virulence? Q5.—Which species of those that infect cattle are melanic? and finally Q6.—Which of the virulence factors are associated with the production of melanin? The answers to these questions will allow us to evaluate the pleiotropic effect of melanin on fungal pathogenesis in bovines.

### 2.2. Naive Search and Early Keywords

Since we do not intend to compare cases, interventions, or results of different quantitative studies, it was not feasible or necessary to use the PICO method (Population, Intervention, Control, Outcome) to establish the conceptual categories of keywords [24]. However, we deconstructed our central research question (i.e., assessing the “Pleiotropic effect of melanin on fungal pathogenesis in bovines”) into four main groups or concepts of keywords. Except for the broad search to compare the relative scientific productivity associated with fungal, bacterial, or viral diseases (Q1), we carried out systematic searches based on combinations of the search terms related to the concepts of fungus, host, melanin, and mycoses. We performed a non-systematic exploration of the bibliography to construct an initial list of search terms that fit into the proposed groups of concepts.

### 2.3. Obtaining the Final Keyword List

We use the ‘litsearch’ R package [22] to identify additional potential search words derived from the early keywords proposed in the previous section. The list obtained was cleaned manually and any incongruous or imprecise terms were removed. The definitive list of search terms is presented in Table 1.

### 2.4. Bibliographic Main Searches in Specialized Databases

We pooled the terms associated with our groups of concepts according to each question in search strings based on Boolean operators. To improve accuracy, we restricted our searches so that search terms appeared in titles and abstracts, and only searched three academic databases: Scopus, Web of Science, and PubMed (Table 2).

The required syntax varies slightly depending on the employed database, so search strings need to be adjusted accordingly. We chose the PubMed, Scopus, and Web of Science platforms because they can be used as primary search systems and are particularly well-suited for evidence-based synthesis in the form of reviews [25]. We conducted these searches in January 2022 and restricted them to articles in English. We report the queries applied to the three data bases and the number of items returned in Table 2.

### 2.5. Creation and Processing of the Corpus

We consolidated the files (*.*bib) that resulted from the searches from each database to confirm and process the corpus with the ‘quanteda’ R package [23]. We automatically removed duplicate records and compiled the year, title, summary, and reference lists for each article in an Excel file. Afterwards, we filtered the articles based on the manual or automatic application of inclusion criteria (Table 3). The selected articles were read and presented as narrative synthesis or tabulated in tables and figures depending on the question.

Since we did not find any references that described melanin mediated mechanisms of pathogenic evasion by fungi in cattle (*Bos taurus*), we evaluated if some immunologic mechanisms referred to for other mammals are transferable via homology to cattle. For this, we extracted the name of the host (model, i.e., humane, murine etc.) gene whose expression affects the immune response to fungal infection from selected publications. Then we searched for the amino acid sequence and verified the metabolic pathway of origin in the Kyoto Encyclopedia of Genes and Genomes (KEGG) [26]. Orthologous genes in cattle with a homology (identity) equal to or greater than 75% were afterwards searched for reciprocal BLAST searches performed in pBLast [27].

## 3. Results

### 3.1. Patterns of Research

**Figure 1:**
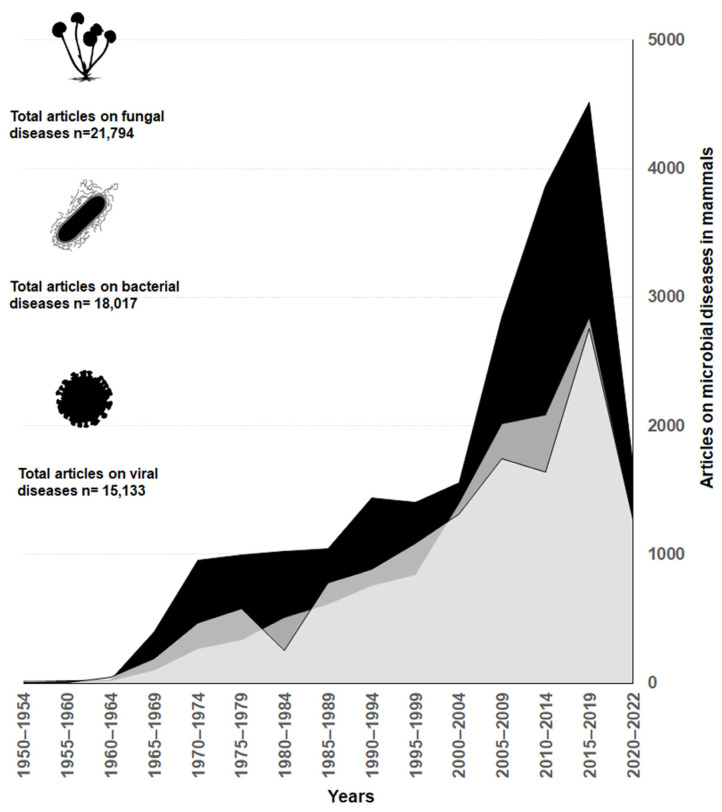
Number of scientific papers studying the three main pathogen groups that infect cattle. The black polygon corresponds to fungi, the intermediate grey polygon to bacteria and the light grey polygon to viruses. The results are based on an exploratory search in PubMed using the search terms available in the Supplementary Material.

Research of cattle mycoses have advanced faster compared to studies of diseases caused by other microorganisms, such as bacteria and viruses. Overall, mycoses research produced 40% of the articles, while research on bacterial and viral infections represented 32 and 28%, respectively. Starting in the 2000s, there is a marked increase in publications related to fungi diseases compared to bacteria and viruses (Figure 1)

### 3.2. Diversity of Cattle Fungal Disease

Based on our analysis of nearly 1500 articles on pathogenic fungi and mycosis in cattle, we identified 107 species of fungi causing mycosis in cattle, distributed across five fungal divisions (Ascomycota, Basidiomycota, Microsporidia, Mortierellomycota, and Mucoromycota). Among these divisions, Ascomycota was the best represented, comprising 84 species. The genera with the highest number of species associated with cattle mycosis were *Candida* (12 species), *Aspergillus* (9 species), *Malassezia* (6 species), and *Trichophyton* (5 species). The detailed diversity of fungi that cause mycosis in bovines is available in Figure S1 and Table S1. The taxonomy used in both is according to Mycobank [16,17]. The Figure S1 is an interactive figure created in Krona [28], arrangement of concentrically nested taxonomic hierarchies from the phylum in the center to the genus in the outer ring.

### 3.3. Virulence

Each of the 107 species of fungi referred to as pathogenic can be indicated as the etiolant agent of one or more of the 18 (common) mycoses presented in Table 4. A comprehensive list is available in the Supplemental Material (Table S1). At least 40 species are predominant or frequent etiolant agents of any of these diseases. Of the pathogenic fungal species of cattle, 25% biosynthesize melanin (Table 5). Table 6 presents three examples of bovine molecular targets inferred via homology and that are associated with metabolic pathways affected by melanin from pathogenic fungi in other model animals (i.e., house mouse and human).

## 4. Discussion

The global trend of fungal diseases being relatively less researched compared to other groups of pathogenic microorganisms has been repeatedly mentioned in the literature [2,3]. However, our review reveals that this is not the case in regards to diseases affecting cattle. On the contrary, the evidence presented in Figure 1 shows that the number of articles on fungal diseases is considerably higher than the ones for bacteria and viruses. Thanks to the scientific progress, we now have a better understanding of how specific mycoses affect livestock welfare and production. Some mycoses, such as those causing mycotic abortions and mastitis, have been extensively studied [57,58,59,60,61].

### 4.1. Diversity of Pathogenic Fungi and Mycoses in Cattle

Rapid evolutionary changes are often associated with antagonistic interactions, such as coevolution between host and pathogen [62]. By definition, pathogens affect the host’s fitness, inducing selection for improved defense mechanisms. Conversely, the improvement of the host’s defense mechanisms will lead to the establishment of more efficient evasion mechanisms by the pathogen [63]. If this antagonism persists for a sufficiently long period, iterative cycles of adaptation and counter-adaptation become an efficient evolutionary force [64]. Numerous examples of rapid evolutionary responses during host-pathogen coevolution exist [65], and resistance to fungal infection in animal hosts could be one of them, although this does not necessarily happen symmetrically.

Some authors hypothesized that the optimization of immune defenses against fungi, including homeothermy, has shaped mammalian evolution [66]. However, it is possible that some virulence factors of pathogenic fungi were acquired as an adaptive response to evade predation or protect against detrimental conditions (i.e., the accidental pathogen hypothesis) [5]. For instance, it is believed that under certain environmental conditions, the extracellular polysaccharide capsule protects *Cryptococcus* cells from phagocytosis by amoeboid protozoa [67]. Similarly, the deposition of melanin in the cell wall of *Cryptococcus* protects against thermal and oxidative stress from solar radiation [68]. However, the presence of a capsule and the ability to produce melanin are also two potent virulence factors that make this species a formidable mammalian pathogen.

Compared to bacterial or viral diseases, Systemic fungal diseases are relatively infrequent in non-immunocompromised mammals [66,69]. Mammals have evolved an increased resistance to fungal infections through adaptive immunity [70]. This enables mammals to discriminate between self and non-self and respond rapidly to current and future invaders [71]. Mammals, being homeothermic vertebrates, possess highly sophisticated innate and adaptive immune systems [72]. Mammalian pathogenic fungi must overcome challenges such as surviving within the internal temperatures of mammals, tolerating slightly alkaline environments, adhering to host tissues, and evading host immune responses [73]. As a result, only a small percentage of the described fungal species are pathogens of mammals.

The current richness of fungi is estimated to be between 1.5 million and 5.1 million species [74], with over 100,000 species and 18,000 genera described. Approximately 625 species have been reported as vertebrate pathogens [61]. Around 325 species of pathogenic fungi have been documented in humans [75], and an even smaller number in other mammals. Only a handful of species are common infectious agents. In this context, it is evident that the ability of fungi to infect mammals is a rare skill [75].

Fungi commonly cause mammal diseases through three mechanisms: allergies, intoxication by mycotoxins or mycoses [76]. Although this is a relatively small group of species, the classification of clinically significant fungi and the diseases they cause have been challenging [77]. In the veterinary literature, mycoses are often classified based on various criteria, such as the anatomical area of the host affected by the mycosis (cutaneous, subcutaneous, or systemic), the predominant causative agent (e.g., Aspergillosis caused by the genus *Aspergillus*), the route of acquisition (exogenous or endogenous), epidemiology, or based on virulence (i.e., opportunistic or commensal). Additionally, specific names are used in animal production, such as fungal abortions and fungal mastitis [78].

During the elaboration of this review, we found no consensus for the use of any of these classifications. Therefore, for practical purposes, the mycoses are preliminarily classified here by integrating several available classifications in the literature (Table 2). This classification is not entirely appropriate, as it exhibits a certain degree of overlap among different categories since, given the opportunistic nature of most causative agents of mycoses, it is common for the same pathogen to be involved in different diseases. However, it can be useful for readers to gain an understanding of the wide spectrum of pathogenic fungi and mycoses that can affect cattle.

### 4.2. Main Virulence Factors of Mammalian Pathogenic Fungi

The concept of virulence represents an emergent property observed in all pathogens. It arises from the intricate interplay of multiple intrinsic morphological, physiological, metabolic, and molecular characteristics of both host and pathogen [66,79]. In the case of fungal infections, successful colonization of host tissues and subversion of the host’s immune response are pivotal. The ability to adhere to host tissues and secrete tissue-degrading enzymes, which elude the host’s immune defenses, are crucial factors in fungal pathogenesis [60]. These evasion and resistance mechanisms against the immune response are known as virulence factors. While our understanding of fungal pathogenicity and virulence-related traits has significantly advanced [5,80,81], these studies have primarily focused on a limited number of pathogenic species, predominantly in humans, murine models (e.g., laboratory mice *Mus musculus*) [82], and invertebrates (e.g., *Galleria mellonella*) [83]. Unfortunately, the knowledge about fungal pathogenesis and virulence factors in bovine infections is limited.

*Aspergillus*, *Candida*, *Cryptococcus*, *Fonsecaea, Histoplasma*, *Paracoccidioides*, and *Sporothrix* are among the most extensively studied genera of mammalian pathogenic fungi that commonly infect cattle [61]. These fungi possess intrinsic characteristics or virulence factors that enable successful attacks on mammals. For example, *Aspergillus* species demonstrate a remarkable dispersal capacity, tolerance to a wide range of environmental conditions, and the production of potent immunosuppressive and cytotoxic mycotoxins, including aflatoxins, gliotoxin, and ochratoxin. *Aspergillus fumigatus*, like other fungi, possesses dozens of genes that produce secondary metabolites and virulence-associated proteins, facilitating their establishment in stressful or demanding environments such as the mammalian body [60,80,84]. Similarly, *Cryptococcus* exhibits key virulence traits such as tolerance to oxidative stress conferred by melanin production through lactase, the formation of a protective extracellular polysaccharide capsule, and a wide range of thermal tolerance [5,48,81,85,86,87].

In *Cryptococcus neoformans* [88,89] and other pathogenic fungi as *Candida auris* [90], *C. glabrata* [91] the generational aging play a role in the virulence increasing resistance to antifungals and phagocytic attacks. The older phenotypes exhibit thickened cell walls. The fungal cell wall is essential for yeast viability and pathogenesis and its thickening seems to have increased levels of all major components, increased intracellular trafficking as well as the alteration of vacuole morphology and pH homeostasis [92]. Thicker walls could also imply an increase in melanin deposition, an important virulence factor in fungi.

### 4.3. Melanin as a Virulence Factor in Fungal Pathogenesis

Melanin is the generic name for a family of structurally complex, insoluble pigments, ranging in color from dark brown to black [93]. Melanin originates from the polymerization of indolic and phenolic compounds. The structural conformation of melanin has not been established. Its macromolecular nature based on indole precursors and its amorphous properties hinders its crystallographic determination. Melanin is widely represented across all kingdoms, from bacteria to chordates [94,95]. In the fungal kingdom, melanization occurs in all phyla, but proportionally in only a few species. Some fungal species are constitutively melanized, known as dematiaceous, and for them melanization is an obligatory condition. On the other hand, facultative melanized fungi synthetize melanin only during certain stages of their development, in response to specific environmental stimuli (e.g., the presence of phenolic melanin precursors or temperature changes) [93].

Various fungal genera produce melanin. In filamentous fungi, such as *Alternaria*, *Armillaria*, *Aspergillus*, *Auricularia*, *Cladosporium*, *Epicoccum*, *Eurotium*, *Magnapothe*, *Ochroconis*, *Penicillium*, *Phomopsis*, *Sporothrix*, *Stachybotrys*, and *Wangiella*, have been found to contain melanin. Melanin production has also occurred in yeast, including *Candida albicans*, *Cryptococcus neoformans*, *Hormoconis resinae*, and *Kluyveromyces marxianus* [5]. While most assays for melanin production are conducted on strains isolated from the environment, humans, murine, or invertebrates’ models such as *Galleria*, it is noteworthy that approximately 35% of fungal species causing mycosis in livestock are melanic species. Certain melanized fungi are the primary agents responsible for mycosis in cattle (Table 5).

Melanin plays a significant role in the pathogenesis of mammalian fungal agents such as *Aspergillus fumigatus* [54], *Cryptococcus neoformans* [48], *Histoplasma capsulatum* [39], and *Paracoccidioides brasiliensis* [39]. Melanin can be considered the Swiss army knife of fungi. Its presence enhances fungal survival in adverse environmental conditions, providing resistance against radiation and enzymatic degradation. A growing body of evidence suggests that melanin makes fungi more resistant to antifungal compounds and other innate immune mechanisms such as phagocytosis [94,96,97]. Overall, melanin exhibits pleiotropic effects, participating in various evasion mechanisms employed by fungal pathogens during specific stages of host immune responses [5]. The primary virulence mechanism associated with melanin is its ability to prevent damage from reactive oxygen species. Melanin neutralizes free radicals, attenuating oxidative stress that could be generated by the oxidative defenses of the immune system or by the environment [98].

Normally, the recognition of any pathogenic cell by the host triggers a response called oxidative burst generated by the innate immune system [75] Immune effector cells such as macrophages, monocytes, and neutrophils release reactive oxygen species (ROS), including superoxide and peroxide ions, as well as nitric oxide [97]. These chemicals are highly cytotoxic and are usually effective in destroying foreign cells. However, melanin is capable of neutralizing oxidative bombardment, protecting the melanotic pathogenic fungi [75].

Melanin can also sequester antifungal drugs as well as the antifungal secondary metabolites [97] as amphotericin B and caspofungin in humans [39] or Fluconazole in cattle [57]. By being located in the cell wall, melanin keeps antifungal compounds in the extracellular space, preventing their cytotoxic effects. Melanin also strongly bind to peptides, immobilizing them, which is a convenient skill for preventing or masking complement deposition on the cell surface [99]. Fungal melanin by itself is an immunogen capable of triggering antibody release in murine models [100]. Resistance to antimicrobial agents has significant implications for morbidity, mortality, and healthcare expenses, as resistant strains are responsible for the majority of infections in both animals and humans [101,102]. However, research on resistance to antifungal agents has historically received less attention than studies focused on understanding antibacterial resistance [103]. Several factors contribute to this disparity. The principal reason for this is the relatively recent recognition of fungal agents as significant pathogens in animals and humans [2,3]. The available information on resistance to fungi in Cattle seems scarce. There are a limited number of antifungal agents licensed for use in animals; however, more and more of those available for the treatment of mycoses in humans are used by veterinarians [104]. In this context, it is certainly to be expected that with the increased use of different antifungal drugs, the number of clinical fungal pathogens that become resistant to these drugs will increase [105].

The melanin of the fungus *Fonsecaea monophora* inhibits the expression of the i-NOS gene in activated mouse macrophages, regulating the production of nitric oxide (NO) and allowing the fungus to avoid oxidative burst [56]. The release of NO by effector cells of the immune system is a consequence of interferon type II signaling associated with macrophage activation. The release of reactive oxygen species by activated macrophages is one of the principal immune mechanisms used to inhibit the propagation of *F. monophora* [56]. This behavior has also been observed in the related species *F. pedrosoi* [34] and in other fungi such as *Cryptococcus neoformans* [106], *Histoplasma capsulatum* [107], and *Sporothrix schenckii* [108].

In *Cryptococcus neoformans*, those phenotypes that produce melanin faster are more-frequent targets of nonlytic exocytosis [109]. Nonlytic exocytosis is a phenomenon where previously internalized microbes are extruded from host phagocytes, allowing the survival of both cell types [110]. The rapid melanin kinetics is mediated by the laccase enzyme, but its influence on nonlytic exocytosis was unknown. The recent experimental evidence suggests that is probably due to higher laccase production, because fungi lacking this enzyme are nonlytically exocytosed less often [109]. This mechanism happens not only in the interactions of *Cryptococcus* spp. and other fungal cells with phagocytes, spanning diverse organisms, including mammalian, avian, and fish macrophages, but also in ameboid predators [67]. So, the laccase can be recognized as a novel fungal virulence factor [109,111]. The discovery that laccase could affect nonlytic exocytosis adds one more melanin-independent role for this crucial enzyme in fungi virulence.

The melanin of *Aspergillus fumigatus* inhibits the assembly and activity of the vacuolar ATPase, a proton pump dependent on ATP type V present in the alveolar phagocytes of the host, preventing the acidification of phagolysosomes and contributes to the pathogenicity of *A. fumigatus* [54]. Additionally, the melanin in the conidia of *A. fumigatus* inhibits the intrinsic apoptosis pathway after phagocytosis by macrophages [112].

In *Histoplasma capsulatum*, melanin induces the formation of granulomas inside mammalian macrophages. Hypoxia-inducible factor 1α (HIF-1α) Infection regulates *H. capsulatum* in mammals, being a key player in macrophage-mediated innate immunity. HIF-1α creates a hostile environment for yeast cells within macrophages by disrupting the pathogen’s ability to induce host cell autophagy [55].

During this review, the absence of publications describing fungal pathogenic evasion mechanisms in cattle (*Bos taurus*) suggests an asymmetry in knowledge production. Model animals such as mice or humans are often better studied, leading to a bias that constantly affects various knowledge domains. However, this bias can be significantly and rapidly reduced through the creative utilization of bioinformatic methodologies. The V-type proton ATPases, the HIF-1-alpha and the Nitric Oxide Synthase, inducible (iNOS) are examples of bovine molecular targets inferred via homology and that are associated with metabolic pathways affected by melanin from pathogenic fungi *Aspergillus fumigatus* [54], *Histoplasma capsulatum* [55] and *Fonsecaea monophorain* [56] from other model animals (i.e., house mouse and human). This is a technique that requires further exploration and experimentation, but we feel it should be addressed very immediately.

## 5. Conclusions

Understanding how melanin affects the virulence of pathogenic fungi is a priority issue in veterinary medicine and animal production. Melanin biosynthesis increases the survival of bovine pathogenic melanic fungi. Melanin confers resistance under environmental (non-pathogenic) conditions, but also virulence to pathogenic fungi in the host. Melanin generates fungus virulence in multiple ways, including by neutralizing reactive oxygen species, sequestering antifungal secondary metabolites, and blocking phagocytosis. However, we still do not know much about its potential as a virulence factor. Based on the evidence presented here, it is clear that despite its relevance in veterinary medicine and animal production, it is still necessary to investigate several aspects of fungal pathogenesis in cattle. Such is the case of virulence mechanisms in which melanin is involved or possible pathways of metabolic regulation of fungal pathogenesis based on biotechnologies aimed at limiting or blocking melanogenesis.

A growing body of research is oriented towards identifying molecular targets associated with melanogenesis. These studies primarily utilize murine and human models. As evidenced here, we can extend this information to different taxonomic groups, such as bovines. For this, it is advisable to generate precise information on the metabolic routes that the host activates in response to the fungus so we can establish homologies efficiently. Studies based on bioinformatics tools allow the generation of synthetic knowledge quickly and consistently with the available evidence, providing a better understanding and management of diseases in which melanin is the main virulence factor.

## Figures and Tables

**Table 1:** Key concepts and their associated search terms used in three online data bases.

Core Concept	Associated Search Terms
Fungi	aspergillus OR candida OR cryptococcus OR enterocytozoon OR exophiala OR fonsecaea OR fungal OR fungi OR fungus OR histoplasma OR paracoccidioides OR penicillium OR sporothrix OR talaromyces OR trichophyton OR yeast
Host	bovid OR bovine OR bovino OR bullocks OR bulls OR calves OR cattle OR cow OR dairy OR livestock
Melanin	melanin OR melanogenesis OR melanotic
Mycosis	aspergillosis OR blastomycosis OR candidiasis OR chromoblastomycosis OR coccidioidomycosis OR cryptococcosis OR dermatophytosis OR epizootic OR abortions OR keratomycosis OR lymphangitis OR maduromycosis OR mastitis OR mucormycosis OR mycetoma OR mycotic OR mycosis OR mycoses OR paracoccidioidomycosis OR phaeomycosis OR pithomycotoxicosis OR pythiosis OR ringworm OR sporotrichosis OR zygomycosis

**Table 2:** Research questions and respective searches keywords used in three online data bases.

Question	Keywords String	Data Base	Processed Articles	
Q1	“fungal AND disease AND cattle”	PubMed	21,794 18,017 15,133	54,944
	“bacterial AND disease AND cattle”			
	“viral AND disease AND cattle”			
Q2–Q4	“associated with the fungi concept” AND “associated with the host concept” AND “associated with the concept mycosis”	PubMed Scopus WoS	417 758 238	1413
Q5–Q6	“associated with the fungi concept” AND “ “associated with the concept melanin”	PubMed Scopus WoS	187 128 120	435

**Table 3:** Articles inclusion criteria to conform the corpus.

Criteria		Type
1	At least one of the keywords associated with each search concept present in the title or abstract	Automatic
2	The article is not duplicated within the corpus	Automatic
3	The article is in English	Automatic
4	It was selected by context in R (questions Q5–Q6 only) associated with one of the connecting words, established	Automatic
5	The article contains some unusual term in capital letters (we did this with the help of the “keep tokens” function and the regular expression “[^[:UPPER:]^]” in R, only questions Q2–Q4)	Automatic
6	Explicitly mentions a pathogenic fungal species or describes a melanin-associated virulence mechanism	Manual

**Table 4:** Main mycoses encountered in bovines and their causative agents. Taxonomy and references available in Table S1.

Mycosis Type	Mycosis	Predominant Mycosis-Causing Agent
Cutaneous, superficial, ringworm or dermatophytosis	Pheomycosis	*Alternaria alternata*
	Epizootic Lymphangitis	*Trichophyton verrucosum*
	Mycetoma or maduromycosis	*Cochliobolus spiciferus*
		*Curvularia geniculata*
		*Drechslera rostratum*
		*Madurella mycetomatis*
		*Pseudallescheria boydii*
	Pythiosis	*Pythium insidiosum*
	Keratomycosis	*Mortierella wolfii*
	Ringworm	*Cladophiolophora bantiana*
		*Microsporum canis*
		*Trichophyton mentagrophytes*
Subcutaneous	Chromoblastomycosis	*Fonsecaea monophora*
		*Fonsecaea pedrosoi*
	Sporotrichosis	*Sporothrix schenckii* and another ssp.
Systemic, primary, internal, disseminated or deep	Cattle Mycotic abortion	*Aspergillus fumigatus*
		*Aspergillus nidulans*
		*Candida albicans*
		*Emericella nidulan*
		*Lichtheimia corymbifera*
		*Mortierella wolfii*
		*Rhizomucor pusillus*
		*Rhizopus arrhizus*
	Aspergillosis	*Aspergillus fumigatus* and another ssp.
	Blastomycosis	*Blastomyces dermatitidis*
	Candidiasis	*Candida albicans* and another ssp.
	Coccidioidomycosis	*Coccidioides immitis*
	Cryptococcosis	*Cryptococcus neoformans*
	Histoplasmosis	*Histoplasma capsulatum*
	Fungal mastitis	*Aspergillus fumigatus*
		*Aureobasidium pullulans*
		*Candida albicans* and another ssp.
		*Candida parapsilosis*
		*Cryptococcus neoformans*
		*Prototheca zopfii*
		*Trichosporon mucoides*
	Paracoccidioidomycosis	*Paracoccidioides brasiliensis*
	Zygomycosis or Mucormycosis	*Syncephalastrum racemosum*
		*Cunninghamella bertholletiae*
		*Saksenaea vasiformis*

**Table 5:** Major mammalian melanistic fungal pathogens infecting cattle, full list of species found in this review available in Table S1.

Division	Species
Ascomycota	*Aureobasidium pullulans* [29]
	*Alternaria alternata* [30]
	*Exserohilum rostratum* [31]
	*Cladophialophora bantiana* [32]
	*Exophiala dermatitidis* [33]
	*Fonsecaea pedrosoi* [34]
	*Aspergillus flavus* [31]
	*Aspergillus fumigatus* [33]
	*Aspergillus nidulans* [35]
	*Aspergillus niger* [36]
	*Aspergillus terreus* [37]
	*Paecilomyces variotii* [38]
	*Blastomyces dermatitidis* [39]
	*Histoplasma capsulatum* [39]
	*Paracoccidioides brasiliensis* [39]
	*Trichophyton rubrum* [40]
	*Yarrowia lipolytica* [41]
	*Candida albicans* [39]
	*Candida parapsilosis* [42]
	*Saccharomyces cerevisiae* [43]
	*Fusarium oxysporum* [44]
	*Sporothrix schenckii* [39]
Basidiomycota	*Malassezia furfur* [45]
	*Malassezia obtusa* [46]
	*Malassezia pachydermatis* [47]
	*Malassezia sympodialis* [46]
	*Cryptococcus neoformans* [48]
	*Papiliotrema laurentii* [49]
	*Trichosporon asahii* [50]
	*Ustilago maydis* [51]
Mortierellomycota	*Actinomortierella wolfii* [52]
Mucoromycota	*Lichtheimia corymbifera* [53]
	*Mucor hiemalis* [53]
	*Rhizopus arrhizus* [53]
	*Rhizopus microsporus* [53]

**Table 6:** Examples of knowledge transfer by homology of orthologous genes.

**Fungi**	*Aspergillus fumigatus*	*Histoplasma capsulatum*	*Fonsecaea monophora*
**Model**	*Mus musculus*	*Homo sapiens*	*Mus musculus*
**Way**	Proton-dependent phagolysosomal acidification type V ATPases	Expression of Hypoxia-inducible Factor 1HFI-1	Type II interferon signaling associated with macrophage activation
**Target**	V-type proton ATPases	HIF-1-alpha	Nitric Oxide Synthase, inducible (iNOS)
**Identity *B.taurus***	91%	94%	88%
**Source**	[54]	[55]	[56]
**KEGG link**	KEGG T01008: 338038	KEGG T01008: 281814	KEGG T01008: 282876

## Data Availability

The data presented in this research are available within the main text of the article or in the Supplemental Material.

## Supplementary Materials

The following supporting information can be downloaded at: https://www.mdpi.com/article/10.3390/jof9090929/s1, Figure S1: Diversity of fungi that cause mycosis in bovines Table S1: Diversity of pathogenic fungi of cattle.
