# Supplementary material for: Fungi’s Swiss Army Knife: Pleiotropic Effect of Melanin in Fungal Pathogenesis during Cattle Mycosis
Source: J Fungi (Basel). 2023 Sep 15;9(9):929. doi: 10.3390/jof9090929 (PMC10532448; doi:10.3390/jof9090929)
Supplement: Supplementary file 1 [file jof-09-00929-s001.zip › TableS1_FigureS1/Figure S1.html]

Javascript must be enabled to view this page.

magnitude

Pathogen

 105

 1

 1

 1

 1

 1

 82

 6

 4

 3

 1

 1

 1

 1

 1

 1

 1

 1

 1

 1

 1

 32

 14

 10

 1

 2

 2

 5

 2

 2

 2

 1

 1

 15

 11

 9

 2

 1

 1

 3

 2

 1

 3

 3

 1

 2

 31

 31

 23

 12

 2

 2

 1

 1

 3

 1

 1

 2

 2

 1

 1

 1

 1

 2

 1

 1

 1

 1

 1

 1

 13

 9

 1

 1

 3

 2

 1

 1

 1

 1

 1

 2

 1

 1

 1

 1

 1

 1

 1

 3

 3

 1

 1

 1

 16

 7

 3

 2

 2

 1

 1

 3

 3

 1

 2

 1

 1

 1

 1

 1

 1

 1

 6

 6

 6

 6

 1

 1

 1

 1

 1

 1

 1

 1

 2

 2

 2

 2

 1

 1

 4

 4

 1

 1

 1

 3

 3

 1

 2
