# Supplementary material for: Fungi’s Swiss Army Knife: Pleiotropic Effect of Melanin in Fungal Pathogenesis during Cattle Mycosis
Source: J Fungi (Basel). 2023 Sep 15;9(9):929. doi: 10.3390/jof9090929 (PMC10532448; doi:10.3390/jof9090929)
Supplement: Supplementary file 1 [file jof-09-00929-s001.zip › TableS1_FigureS1/Suplementari captions.docx]

**Suplementari captions**

**Figure S1**. Diversity of fungi that cause mycosis in bovines. The taxonomy according to Mycobank [16,17]. Created in Krona [28]. Arrangement of concentrically nested taxonomic hierarchies from the phylum in the center to the genus in the outer ring.

**Table S1.** Pathogenic fungi of cattle
