# Supplementary material for: Fungi’s Swiss Army Knife: Pleiotropic Effect of Melanin in Fungal Pathogenesis during Cattle Mycosis
Source: J Fungi (Basel). 2023 Sep 15;9(9):929. doi: 10.3390/jof9090929 (PMC10532448; doi:10.3390/jof9090929)
Supplement: Supplementary file 1 [file jof-09-00929-s001.zip › TableS1_FigureS1/Table S1.docx]

**Table S1.** Pathogenic fungi of cattle

| **Taxa** | **References** |
| --- | --- |
| Ascomycota |  |
| Dothideomycetes |  |
| Botryosphaeriaceae |  |
| *Neoscytalidium dimidiatum* | [1] |
| Pleosporaceae |  |
| *Alternaria alternata* | [2] |
| *Curvularia geniculata* | [1] |
| *Exserohilum rostratum* | [3] |
| Saccotheciaceae |  |
| *Aureobasidium pullulans* | [2] |
| Torulaceae |  |
| *Torula mucilaginosa* | [4] |
| Eurotiomycetes |  |
| Herpotrichiellaceae |  |
| *Fonsecaea pedrosoi* | [5] |
| Ajellomycetaceae |  |
| *Blastomyces dermatitidis* | [6] |
| *Histoplasma capsulatum* | [7] |
| *Paracoccidioides brasiliensis* | [8] |
| Arthrodermataceae |  |
| *Arthroderma tuberculatum* | [2] |
| *Microsporum canis* | [2] |
| *Microsporum gallinae* | [2] |
| *Nannizzia gypsea* | [2] |
| *Nannizzia nana* | [2] |
| *Trichophyton benhamiae* | [4] |
| *Trichophyton mentagrophytes* | [4] |
| *Trichophyton rubrum* | [4] |
| *Trichophyton simii* | [4] |
| *Trichophyton verrucosum* | [2] |
| Aspergillaceae |  |
| *Aspergillus clavatus* | [2] |
| *Aspergillus flavus* | [1] |
| *Aspergillus fumigatus* | [1] |
| *Aspergillus nidulans* | [1] |
| *Aspergillus niger* | [2] |
| *Aspergillus ochraceus* | [2] |
| *Aspergillus rugulosus* | [1] |
| *Aspergillus sydowii* | [2] |
| *Aspergillus terreus* | [1] |
| *Penicillium herquei* | [2] |
| *Penicillium italicum* | [2] |
| Herpotrichiellaceae |  |
| *Cladophialophora bantiana* | [2] |
| *Exophiala dermatitidis* | [3] |
| *Exophiala jeanselmei* | [3] |
| Onygenaceae |  |
| *Chrysosporium keratinophilum* | [2] |
| *Chrysosporium tropicum* | [2] |
| Thermoascaceae |  |
| *Paecilomyces variotii* | [2] |
| Trichocomaceae |  |
| *Talaromyces funiculosus* | [2] |
| *Talaromyces ruber* | [2] |
| *Thermomyces thermophilus* | [1] |
| Saccharomycetes |  |
| Debaryomycetaceae |  |
| *Debaryomyces hansenii* | [9] |
| Dipodascaceae |  |
| *Saprochaete ingens* | [10] |
| *Yarrowia lipolytica* | [11] |
| Endomycetaceae |  |
| *Trichomonascus ciferrii* | [9] |
| Insertae sedis |  |
| *Cyberlindnera fabianii* | [10] |
| Metschnikowiaceae |  |
| *Clavispora lusitaniae* | [12] |
| Saccharomycetaceae |  |
| *Candida albicans* | [11] |
| *Candida brumptii* | [10] |
| *Candida ethanolica* | [9] |
| *Candida maltosa* | [9] |
| *Candida membranaefaciens* | [10] |
| *Candida parapsilosis* | [12] |
| *Candida pararugosa* | [9] |
| *Candida solani* | [10] |
| *Candida tenuis* | [10] |
| *Candida tropicalis* | [12] |
| *Candida zeylanoides* | [11] |
| *Cyberlindnera jadinii* | [12] |
| *Diutina catenulata* | [11] |
| *Diutina rugosa* | [12] |
| *Kluyveromyces lactis* | [12] |
| *Kluyveromyces marxianus* | [11] |
| *Meyerozyma guilliermondii* | [11] |
| *Ogataea polymorpha* | [10] |
| *Pichia holstii* | [10] |
| *Pichia kudriavzevii* | [11] |
| *Pichia norvegensis* | [10] |
| *Saccharomyces cerevisiae* | [4] |
| *Yamadazyma mexicana* | [9] |
| Wickerhamomycetaceae |  |
| *Wickerhamomyces anomalus* | [10] |
| Sordariomycetes |  |
| Coniochaetaceae |  |
| *Coniochaeta hoffmannii* | [1] |
| Hypocreaceae |  |
| *Sepedonium chrysospermum* | [13] |
| *Trichoderma viride* | [13] |
| Insertae sedis |  |
| *Cephalosporium acremonium* | [13] |
| Microascaceae |  |
| Microascus paisii | [2] |
| *Pseudallescheria boydii* | [1] |
| *Scopulariopsis brevicaulis* | [13] |
| Nectriaceae |  |
| *Fusarium oxysporum* | [2] |
| *Fusarium verticillioides* | [2] |
| *Neocosmospora solani* | [13] |
| Ophiocordycipitaceae |  |
| *Purpureocillium lilacinum* | [2] |
| Ophiostomataceae |  |
| *Sporothrix schenckii* | [14] |
| Sarocladiaceae |  |
| *Sarocladium kiliense* | [2] |
| Stachybotryaceae |  |
| *Stachybotrys chartarum* | [13] |
| Basidiomycota |  |
| Cystobasidiomycetes |  |
| Cystobasidiaceae |  |
| *Cystobasidium minutum* | [11] |
| Malasseziomycetes |  |
| Malasseziaceae |  |
| *Malassezia furfur* | [15] |
| *Malassezia globosa* | [15] |
| *Malassezia obtusa* | [15] |
| *Malassezia pachydermatis* | [15] |
| *Malassezia slooffiae* | [15] |
| *Malassezia sympodialis* | [15] |
| Microbotryomycetes |  |
| Sporidiobolaceae |  |
| *Rhodotorula* spp. | [10] |
| Tremellomycetes |  |
| Cryptococcaceae |  |
| *Cryptococcus bacillisporus* | [10] |
| *Cryptococcus neoformans* | [10] |
| Filobasidiaceae |  |
| *Naganishia albida* | [10] |
| Rhynchogastremataceae |  |
| *Papiliotrema laurentii* | [11] |
| Trichosporonaceae |  |
| *Cutaneotrichosporon cutaneum* | [4] |
| *Trichosporon asahii* | [11] |
| *Trichosporon beigelii* | [10] |
| Ustilaginomycetes |  |
| Ustilaginaceae |  |
| *Ustilago maydis* | [16] |
| Microsporidia |  |
| Microsporea |  |
| Encephalitozoonidae |  |
| *Encephalitozoon bieneusi* | [3] |
| *Septata intestinalis* | [13] |
| Mortierellomycota |  |
| Mortierellomycetes |  |
| Mortierellaceae |  |
| *Actinomortierella wolfii* | [1] |
| Mucoromycota |  |
| Mucoromycetes |  |
| Lichtheimiaceae |  |
| *Lichtheimia corymbifera* | [1] |
| Mucoraceae |  |
| *Mucor hiemalis* | [13] |
| *Rhizopus arrhizus* | [1] |
| *Rhizopus microsporus* | [1] |

**References**

1. Knudtson, W.U.; Kirkbride, C.A. Fungi Associated with Bovine Abortion in the Northern Plains States (USA). *J VET Diagn Invest* **1992**, *4*, 181–185, doi:10.1177/104063879200400211.

2. *Emerging and Epizootic Fungal Infections in Animals*; Seyedmousavi, S., De Hoog, G.S., Guillot, J., Verweij, P.E., Eds.; Springer International Publishing: Cham, 2018; ISBN 978-3-319-72091-3.

3. Santín, M.; Fayer, R. Microsporidiosis: Enterocytozoon Bieneusi in Domesticated and Wild Animals. *Research in Veterinary Science* **2011**, *90*, 363–371, doi:10.1016/j.rvsc.2010.07.014.

4. Gugnani, H.C. Mycotic Infections in Animals in India: An Update. *RVSM* **2021**, *1*, doi:10.25259/RVSM_2_2021.

5. Murthy, R.; Swain, J.P. Concurrent Mycetoma and Chromomycosis. *Indian J Med Microbiol* **2011**, *29*, 437–439, doi:10.4103/0255-0857.90192.

6. Kuria, J.N.; Gathogo, S.M. Concomitant Fungal and Mycobacterium Bovis Infections in Beef Cattle in Kenya. *Onderstepoort J Vet Res* **2013**, *80*, 585, doi:10.4102/ojvr.v80i1.585.

7. Adebiyi, A.I.; Oluwayelu, D.O. Zoonotic Fungal Diseases and Animal Ownership in Nigeria. *Alexandria Journal of Medicine* **2018**, *54*, 397–402, doi:10.1016/j.ajme.2017.11.007.

8. Silveira, L.H.; Paes, R.C.S.; Medeiros, E.V.; Itano, E.N.; Camargo, Z.P.; Ono, M.A. Occurrence of Antibodies to Paracoccidioides Brasiliensis in Dairy Cattle from Mato Grosso Do Sul, Brazil. *Mycopathologia* **2008**, *165*, 367–371, doi:10.1007/s11046-008-9095-2.

9. Hayashi, T.; Sugita, T.; Hata, E.; Katsuda, K.; Zhang, E.; Kiku, Y.; Sugawara, K.; Ozawa, T.; Matsubara, T.; Ando, T.; et al. Molecular-Based Identification of Yeasts Isolated from Bovine Clinical Mastitis in Japan. *Journal of Veterinary Medical Science* **2013**, *75*, 387–390, doi:10.1292/jvms.12-0362.

10. Watts, J.L. Etiological Agents of Bovine Mastitis. *Veterinary Microbiology* **1988**, *16*, 41–66, doi:10.1016/0378-1135(88)90126-5.

11. Zhou, Y.; Ren, Y.; Fan, C.; Shao, H.; Zhang, Z.; Mao, W.; Wei, C.; Ni, H.; Zhu, Z.; Hou, X.; et al. Survey of Mycotic Mastitis in Dairy Cows from Heilongjiang Province, China. *Trop Anim Health Prod* **2013**, *45*, 1709–1714, doi:10.1007/s11250-013-0419-y.

12. Du, J.; Wang, X.; Luo, H.; Wang, Y.; Liu, X.; Zhou, X. Epidemiological Investigation of Non-Albicans Candida Species Recovered from Mycotic Mastitis of Cows in Yinchuan, Ningxia of China. *BMC Veterinary Research* **2018**, *14*, 251, doi:10.1186/s12917-018-1564-3.

13. Bagy, M.M.K. Fungi on the Hair of Large Mammals in Egypt. *Mycopathologia* **1986**, *93*, 73–75, doi:10.1007/BF00437737.

14. Dalis, J.S.; Kazeem, H.M.; Kwaga, J.K.P.; Kwanashie, C.N. Severe Generalized Skin Lesions Due to Mixed Infection with Sporothrix Schenkii and Dermatophilus Congolensis in a Bull from Jos, Nigeria. *Veterinary Microbiology* **2014**, *172*, 475–478, doi:10.1016/j.vetmic.2014.05.014.

15. Duarte, E.R.; Batista, R.D.; Hahn, R.C.; Hamdan, J.S. Factors Associated with the Prevalence of Malassezia Species in the External Ears of Cattle from the State of Minas Gerais, Brazil. *Medical Mycology* **2003**, *41*, 137–142, doi:10.1080/mmy.41.2.137.142.

16. Ivanov, X. Ustilagineous Pneumonia in Cattle; the Spores of Ustilago Maydis as a Pathogenic Factor. *C R Acad Bulg Sci* **1949**, *2*, 49–52.
